# Medical Essays

**Published:** 1826-04

**Authors:** 


*Medical Essays. Essay First.—On the Effects of Intestinal Irritation. Essay Second.—On some Effects of Loss of Blood. Essay Third.—On Exhaustion and Sinking from various Causes.* By MARSHALL HALL, M.D. F.R.S. E. Physician to the General Hospital near Nottingham, &c. &c. 8vo. pp. 96. December, 1825.

We have, on more than one occasion in this Journal, observed, that a medical practitioner, in going his daily round, can rarely give a nosological place or character to one half of the diseases presented to his view! This is a humiliating consideration for the systematic writer—but the fact is not to be disguised. Nothing then can be more beneficial for the inexperienced practitioner, than the publication of Essays on those anomalous affections which are constantly crossing his path, and for an account of which he looks in vain to the manuscript lectures of his teacher, or the systems of medicine in his library.

The author of the little volume before us, has long been known as a most accurate and minute observer of the phenomena of diseases; and of great zeal, we had almost said enthusiasm, in the cause of medical science. We are always glad, therefore, to meet with him in print, as we are sure that the public will be gainers on the occasion.

The effects of intestinal irritation, of exhaustion from loss of blood, and of exhaustion and sinking from other causes, have almost altogether, in Dr. Hall's opinion, escaped the observation of medical writers. But surely this is not the case. Since the doctrines of Mr. Abernethy and the innumerable other chylopoietic writers became fashionable, "intestinal irritation" has been in the mouth of almost every practitioner, and on the tip of every writers' pen. Then again, on the Continent, what is the disease which is not attributed by the school of Broussais to intestinal irritation? We are ready to accord to our author, however, the merit of being among the first to point out some *peculiar effects* of intestinal irritation, especially in the female sex and in the parturient state—and of more accurately delineating these peculiar effects than any preceding writer. But although authors should not have distinctly written on these effects, certainly every observant practitioner must have met with them in the course of his practice, though, like numerous other valuable observations, they are lost to the public at large.

*Essay* 1.—*Intestinal Irritation.* There are some effects, Dr. Hall observes, arising from this source, which, although not of an acute or alarming character, are yet not generally discriminated from some other morbid affections of a totally different nature, and requiring very opposite modes of treatment. One effect of intestinal irritation is a cerebral affection resembling, in many instances, the most acute *phrenitis*—in others, enteritis, peritonitis, or pleuritis. Very frequently two or more of these affections take their rise in succession, the first or second probably ceasing entirely before the subsequent one is established—an event which Dr. Hall thinks has often led to an erroneous idea of inflammatory metastases.

" *The Causes.* The principal cause of this morbid affection is a state of intestinal irritation of some duration, arising from a loaded condition of the bowels or from a scybalous or disordered condition of their contents. But, although the presence of this cause appears essential to the production of the complaint, it is important to remark, that I do not remember to have observed any example of it arising quite spontaneously from this cause *alone*. In every case there has been some superadded cause,—some shock sustained, or some extraordinary effort made on the part of the constitution, to rouse the dormant irritation into effect. Unusual fatigue, exertion, loss of rest, anxiety, or alarm,—a fall, or similar *accident*,—exposure to wet or cold,—any cause of weakness and especially of exhaustion, and particularly the combination of some of these circumstances always attendant on parturition, are the principal exciting causes of this affection. The patient has, in many instances, been subject to indigestion. And he is particularly liable to experience returns of the affection in the same or some other form, until the primary disorder and the consequent debility be finally removed.

"*The Symptoms.* This affection generally begins in the manner of a sudden attack. This attack is usually ushered in by rigor,—indeed by a more distinct and decided rigor than is observed in many cases of inflammation; the rigor is usually soon followed by much heat of surface; with the heat the patient experiences some affection of the head, chest, or abdomen, and, indeed, more or less, of all:—there are vertigo on raising the head, pain, and some morbid impression on the mind,—panting in the breathing,—and fluttering about the heart,—with general hurry, irritability, and restlessness; the tongue is white and loaded: the alvine evacuations are morbid,—dark-coloured, fœtid, and scybalous,—or yellow like the yolk of egg,—or of the appearance of yeast; the urine is turbid and frequently deposits a copious sediment." 8.

A more accurate idea of the symptoms is next conveyed by the relation of some cases. We shall briefly advert to a few of these. The first was a Mrs. Hawkins, aged 35, to whom Dr. H. was called, who considered her as labouring under peritoneal inflammation, the symptoms of which were so severe as apparently to demand the repeated employment of the lancet and leeches, so that the patient lost nearly 40 ounces of blood. The bowels were freely opened—motions fœtid. All the symptoms were removed on the 3d day, and our author had every hope of a speedy recovery. Early on the fourth day, however, he was urgently summoned to her. She had been seized with severe pain of the head, especially over the eye-brows, attended with pulsation there, and intolerantia lucis of the most severe kind—*countenance pale and sallow*—pulse full and frequent—no faintness or sighing.

"As this case occurred early in my investigation of the effects of intestinal irritation, I hesitated in determining whether the symptoms were such as I had already witnessed in one or two cases arising from that cause, or were indicative of inflammation within the head. I prescribed a draught with thirty drops of the tinctura opii and of the spiritus ammoniæ aromaticus, and called again in an hour and a half—not without anxiety. I was greatly relieved to find my patient better in every respect,—able to bear the light, suffering much less pain, and having enjoyed a comfortable sleep after a night of wakefulness and distress. Aperient medicine was administered, and, after the full evacuation of the bowels, light nourishment and a repetition of the draught with tinctura opii and spiritus ammoniæ aromaticus, whilst a cold lotion was applied to the head. On the succeeding day Mrs. Hawkins was better in every respect, but complained of any noise. On the next day she was comparatively well, only suffering from vertigo on raising the head. From this time the recovery was progressive and uninterrupted, the utmost care being taken to regulate the bowels and the diet." 10.

We have repeatedly seen symptoms indicative of inflammation depend on irritation, and thus lead practitioners astray —for a time. But what would be the consequence if practitioners always, or very often suspected this state of things, and commenced with opiates, &c.? How much greater would be the danger than in the opposite course, of commencing with sanguineous and intestinal evacuations! The great error consists in perseverance with either measure when no advantage is accruing. Dr. Hall remarks on this case, that "the symptoms were, in the second attack, those usually deemed indicative of *phrenitis* in its *most marked form*." Now what is the practitioner to go by, but the symptoms?—How is he to find out the deception but by the failure of the usual means of cure? Is it not the safer plan, when the symptoms are those of inflammation, to begin with depletion?

*Case* 2. A medical gentleman, Mr. Oldknow of Nottingham, had undergone a painful operation, on the anus, whence he had suffered a good deal, for six weeks—being kept low, with restless nights and painful days.

"On the *sixth day*, after describing the pain and suffering he had experienced from the evacuation of the bowel, which contained scybalous fæces, and from the dressing of the wound, he says, 'I now began to experience great soreness and tenderness in the right groin, and down the back of the thigh; *my feet and legs became extremely cold*; but in about an hour's time, by the application of warm flannels, bottles, and friction by the hands, and by taking a small basin of broth, the coldness and aching subsided; *I became generally heated*, and my mouth was clammy; there came on a general *soreness* of the skin, and especially of the eye-balls; my face was heated and flushed; my pulse gradually rose to 96; but I became quite easy, except from a tenderness in the groin. I took soda-water. I attributed this attack, in part, to a little exertion. At two o'clock in the afternoon *the chillness returned*, and was removed towards three, at which time I experienced *throbbing in my temples with headach*, and flying *stitches in my side;* pulse 112. I was bled to sixteen ounces; after which I began to perspire, and felt a little faint; the throbbing of the head ceased, but the pain continued; pulse 116. A little before five o'clock the perspiration had ceased, having been pretty general, but not profuse. I felt very much *overcome*, and general lightness and vertigo on moving; great thirst. At half after six o'clock my *faintness* continues; I am afraid of taking medicine for fear of sickness; I have pain of the head; and if I raise it from the pillow, I feel giddy and confused. My skin is tolerably cool; my thirst continues; I feel very *restless*, and cannot help *sighing;* the *fan* and aromatic vinegar seem to revive me a little; if I doze, my breathing is alternately very prolonged and very rapid; pulse 108; tongue covered with a thin white mucus; the other groin is a little painful. 8 o'clock.—Much the same. I am directed to take a dose of calomel at bed time, and of ol. ricini in the morning; to foment the groins and arms, and omit introducing the dressings.'—On the *seventh day*, the patient dictated the following report. 'In the early part of the night my *head* became *distractingly painful*, for which I applied cold wet cloths to my head, face and neck, with much relief; the *faintness* was very distressing. Between 11 and 12 o'clock I had a large glyster, composed of gruel and oil, which induced two stools, attended by great faintness; I took sal volatile and nourishment. After the second evacuation my head was relieved, but the faintness continued. I dozed at intervals until 3 o'clock, when I took the ol. ricini, with nourishment at short intervals. I had a copious stool at 6 o'clock, and vomited; my headach and faintness were much relieved; I slept comfortably, and perspired profusely. Betwixt 8 and 9 o'clock I had another motion; the pain of my head was quite gone, as well as the faintness, except after any exertion; the stools were passed with ease; the pain of the groins abated, so that the wound was again dressed with lint; my pulse had got down to 92; I continued to take as much nourishment as I could bear. About 12 o'clock I fell into a sound sleep, which continued, with one trifling interruption, until 4, when I awoke in a profuse perspiration, had my linen changed, and felt considerably better. At the present time, 7 o'clock, I am quite free from pain and faintness; but I feel occasionally flushed, and have a strong disposition to perspire; pulse 84," &c. 14.

In this case, the history of the patient, the local affection, and the symptoms, were so completely those of irritation and debility, that we do wonder how any man in his senses could have ordered venesection! A little more depletion, and Mr. O. would have had a regular—or rather an irregular intermittent fever, from which he could only have been rescued by bark and nourishment.

*Case* 3. Mrs. Darley, a young married lady, in the fourth month of pregnancy, and habitually costive, was attacked, after much fatigue in travelling, with pain in the head, for which leeches were applied to the temples. *Second day.* Pain of head more violent, and attended with much throbbing of the temples—pain under the right breast—tightness across the chest—hurried breathing. Twelve ounces of blood drawn, and an efficient aperient administered. On the third and fourth days, the patient was much better. On the *fifth day*, she was again taken worse, after imprudently sitting up. She had the beating of the temples, the tightness of the chest, and the hurried breathing returned, but without cough. (No mention is ever made of the state of the pulse or the temperature of the body, &c. which are irreparable omissions.) Sixteen ounces of blood drawn, "with great relief." The aperient medicine repeated. The patient continued much better during the sixth and seventh days; but in the night of the latter, she was again seized with severe pain and beating of the head—tightness and pain across the chest—violent palpitation of the heart. Twelve ounces of blood drawn, calomel and other aperients given. Relief again followed. On the eighth day a physician was consulted, and prescribed some pilula hydrargyri and an aperient draught. In the night, the former symptoms all returned, with the addition of cough. Eight ounces of blood drawn—"great relief was obtained." *Ninth day.* The physician again ordered ten ounces of blood to be drawn, "with great relief"—an aperient given—a mixture for the cough. *Tenth day.* In the night of this day, all the symptoms returned in a still more aggravated degree, the palpitation and cough being extremely severe. *Eleventh day.* Dr. Hall was called in.

"There were much pain and throbbing of the *head*, which felt benumbed and heavy as if she could not raise it from the pillow; there had been no sleep; the pupils were extremely small, with intolerance of noise and disturbance of any kind;—there were palpitation of the *heart* and sometimes faintness and a feeling of sinking or dying;—there were a sense of tightness across the *chest*, oppression in the breathing, and a peculiar tracheal or laryngeal cough;—some pain in the region of the *uterus* increased by pressure, but no vaginal discharge;—the countenance was usually pale, but sometimes flushed, the tongue extremely loaded, and even black at the back part, the alvine evacuations, on giving purgative medicine, were still, at first, dark-coloured, offensive, and scybalous,—and afterwards offensive and like yeast; the pulse was 120. I was forcibly struck by a general but marked resemblance of this case, to those already given, and to others of the same nature which I had witnessed:—the depleting plan already fully adopted and repeated had proved ineffectual in affording relief; the purgatives hitherto given, were, I believed, inefficient. The plan I proposed was to give efficient purgatives,—to restrain their operation by draughts with tinctura opii and spiritus ammoniæ aromaticus,—to support the strength by means of nourishment given every hour or oftener,—to procure sleep by anodyne enemata,—to guard against exertion or fatigue,—noise or disturbance.—The recovery was uniformly progressive; there was not even one recurrence of the painful attacks; the symptoms gradually disappeared, the pulse becoming natural, the pupils of the natural size, the head and chest being relieved, and the bowels daily but fully moved; quiet sleep, and a good appetite returned; in six days the patient was convalescent; shortly afterwards she bore a long journey home without any ill consequence, and, at the proper time, had a safe delivery." 19.

We agree with Dr. Hall respecting the state of the case, when he was called in—for previous to that period, the symptoms are not properly related. We have no doubt that, after the first bleeding and aperient, an anodyne combined with a purgative would have saved this poor lady—much medical discipline, and much doctor's stuff.

*Case* 4. Mr. Hastie, aged 40, was attacked with symptoms which were, Dr. H. thinks, mistaken for pleuritis. "He was profusely bled, and lost nearly a gallon of blood." At first the pain was mitigated, but it always returned with unremitted violence. Dr. H. was therefore consulted.

"The pain was referred to the *right side*, over the false ribs, and was excruciating on drawing a deep inspiration, but less so on breathing deep a second and third time: the pulse was about 86; the tongue white and loaded. As blood-letting had been fully tried without effect, and as I entertained the opinion that the pain was symptomatic of intestinal irritation, rather than inflammatory, I prescribed a brisk purge, the operation of which was to be followed by the ammoniacal opiate draught. The motions were dark and fetid. This plan was repeated daily, with a strict attention to nourishment:—the pain *moved* to the *right breast*, and, afterwards, to the *back*, and was extremely severe on drawing a deep breath. By pursuing this mode of treatment, the pain gradually subsided; and on the 9th day of my visits, and 25th of the disease, it was nearly gone, and the pulse natural. During the continuance of the pain, much relief was obtained by the application of a liniment and fomentation.

"At one time, the pulse was 120 from *mental agitation*, and continued frequent during several days; and there were hurry and agitation from any sudden noise, as that of the knocker, or of any thing falling on the floor,—a tendency to talking and delirium, restlessness and picking of the bed-clothes,—heat and perspiration during sleep. The operation of the medicine often induced faintishness; the face and hands were blanched. The purge was given daily, the draught with tinctura opii and spiritus ammoniæ aromaticus, three times a day; the liniment and fomentation when required for pain; and nourishment in small quantity almost every hour. The recovery was progressive, and without any untoward circumstance, except the effects of mental agitation just mentioned. The patient, however, continued to labour under derangement of the general health for some time." 22.

To our apprehension it appears very probable that the pain "over the false ribs," increased by a very deep inspiration, with a pulse at 86, was indicative of its seat being muscular—in fact, that it was rheumatic, which did by no means require the loss of a gallon of blood, and would have done well under very mild treatment, or perhaps no treatment at all. The pain moved to the right breast, and afterwards to the back—and, with submission to Dr. Hall, we cannot clearly see the proof of its source in intestinal irritation. The nervous symptoms which followed were, in our humble opinion, more owing to the ultra depletion employed, than to any thing particular in the primæ viæ.

Having thus given some idea of the general symptoms and the local complications, in the cases detailed, our observant author goes on to a more special examination of the latter phenomena.

"The affection of the *Head* consists of the most acute pain, the greatest intolerance of light, and sound, and the severest form of vertigo, wakefulness, and distress, and sometimes even delirium, and the pupils of the eyes are often extremely contracted.

"The affection of the *Chest* is denoted by severe and acute pain of some part, which is apt to vary its situation, passing from one side to the other, or to the *back*, or occupying a situation higher up or lower down; this pain checks a deep inspiration, and even the ordinary breathing, to which it imparts a character of difficulty and anxiety.

"When the *Abdomen* is affected, there are acute pain, and great tenderness under pressure, in some part, or more or less generally diffused. The attack and situation of the pain is such, in some instances, that the case is with difficulty distinguished from *gall-stones*, though it more generally resemble *enteritis*.

"When the *Heart* is the seat of this affection, there are violent and terrific attacks of palpitation,—and the course of the carotids and even of the abdominal aorta, is sometimes the seat of violent pulsation or throbbing.

"All these affections are apt to occur in sudden attacks, and to recur in paroxysms,—perhaps varying their form,—and exciting great alarm in the patient and his friends, who usually dispatch a hurrying message, to the medical attendants.

"*The Diagnosis.* I now come to the most important and difficult part of my undertaking. The preceding cases are sufficient to establish the fact that there *are* attacks which *resemble* inflammation of the head, chest, or abdomen, and yet which are totally different in their nature. This fact is, of itself, highly important. And if I should fail in giving sufficient diagnostic marks of these morbid affections, it will still be of the utmost moment to know, that the distinction is absolutely essential to the adoption of an appropriate mode of treatment; and that whilst we appeal to future experience to render the diagnosis more complete, the peculiarities of each case must be carefully seized in order to supply the deficiency of the general rule.

"I would first observe that the attack from intestinal irritation, is, in general, more *sudden* than that of inflammation, which is generally formed somewhat more gradually. This circumstance must, therefore, be cautiously inquired into, and may *assist* the diagnosis.

"I believe, too, that the seizure in the former case is attended by *more distinct rigor*, and afterwards by *greater heat*, than in the latter.

"The case of intestinal irritation affects, in a marked degree, *more organs at once*, than that of inflammation, which is usually confined, at first, at least, to one.

"The state of the *tongue* and the condition of the *alvine evacuations* are far more marked by disorder, and the latter are far more offensive, in attacks from intestinal irritation than in cases of inflammation.

"The affection of the *Head* from intestinal irritation comes on suddenly, is formed all at once, and is attended by great restlessness, suffering, and distress. In *phrenitis*, the disease is usually formed somewhat more gradually; the patient has been subject to pain of the head perhaps for some days or even longer; he complains less; or at least there is less urgent distress,—less distress of a general kind; the pain may be very severe, although it is more frequently rather obscure; the intolerance of light and sound is less urgent; the rigor, and subsequent heat, and the attack in general are less marked; the patient is not so soon *relieved* by remedies, and the tongue and alvine evacuations are less morbid. In the attack of affection of the head from intestinal irritation, the patient is *relieved* perhaps completely if the lancet be employed, but the attack soon recurs with equal or greater violence; in phrenitis, the relief is seldom so complete, the interval of ease so long, or the return so marked,—the pain is *diminished*, perhaps, but gradually resumes its former violence, unless active measures be interposed.

"When the *Chest* is affected from intestinal irritation, the pain is severe and acute, and increased by a full inspiration,—if the inspiration be repeated, however, a second and a third time, the increase of the pain is less and less; the situation of the pain varies; there is no cough,—and no crepitus on making a full expiration. In all these respects the case differs from inflammation. The remarks already made respecting the relief from remedies, the tendency to a sudden recurrence of the pain, &c. in cases of affection of the head, apply equally here." 26.

After some recapitulatory observations on the history or course of these affections, Dr. Hall goes on to some other points of considerable interest, which we cannot convey in a better form than his own words:—

"It must have fallen to the lot of many physicians to witness very severe morbid affections, immediately consequent upon causes which appeared totally inadequate to the production of such effects.—A slight blow, or a trifling fall has appeared to induce serious and alarming indisposition. The truth is that there was already a disordered and loaded state of the bowels,—dormant until roused into effect by the accident.—A lady about 50 years of age, fell a few steps down stairs; she got up however and walked to the sofa; in a short time she was taken with chilliness, succeeded by heat of skin and the most intolerable pain of the head and sensibility to light, noise, &c. She soon recovered on taking active purges alternated with the ammoniacal anodyne draught.

"2dly. Every physician must also have observed cases of apparent inflammation, which did not pursue the *usual course* of inflammation,—probably yielding sooner than is generally observed,—or receding altogether, and recurring in paroxysms. This course of the disorder is noticed in several of the cases given in this essay.

"3dly. The case is often *relieved*, perhaps, but obstinately refuses to *yield* to the lancet, recurring with great, if not increased violence,—in a manner not observed in cases of inflammation.

"4thly. In other instances the local affection ceases,—perhaps for a day or two even,—and then recurs, only attacking some distant part. In these cases it has often been thought that there had been a *metastasis* of the former local affection, whilst, in fact, the *cause of both* remaining unremoved, has exerted its influence through a different channel of sympathy and upon another organ.

"5thly. In the same manner we sometimes observe cases apparently involving inflammation of more vital organs than one, at the same time. Such cases may certainly occur. But it is my present object to show that appearances may be deceptive, and that the case may be different from inflammation, and dependant on a disorder remote from the parts affected.

"6thly. In the last place, there have been many cases in which the expected traces of morbid anatomy,—the effects of inflammation within the head, chest, or abdomen,—have been absent altogether. This observation has been fully illustrated, in regard to the *brain*, in the recent works of M. M. Parent Duchatelet, and Martinet,* and of M. Hebreart ;† all these authors have noticed cases in which the *symptoms* of phrenitis existed, and yet not a trace of the effects of inflammation on dissection. The view which has been given of the effects of intestinal irritation may assist us in explaining an event which must have been witnessed by all who have in any degree pursued the study of morbid anatomy." 30.

*Treatment.* This is comprised in a very few words—free

* "De l'Arachnitis, pp. 24, 25."

† "Annuaire Med. Chir. See Dr. Johnson's Review, No. X. pp. 435, 438; see also No. VIII, p. 731."

evacuation of the bowels—soothing by anodynes—light nourishment—and certain local remedies.

Dr. Hall makes some judicious observations on the use of the lancet. He remarks, that could our diagnosis be always early and certain, then the lancet might probably be dispensed with altogether; but there are two reasons why we are not to discard this instrument—first, because what was originally *irritation*, may end in *inflammation*—secondly, because we may be mistaken in the diagnosis. It will, therefore, be prudent to bleed with caution, for the sake of safety, whilst we enforce the other and more specific modes of treatment.

The local applications are chiefly cold lotions to the head—stimulating liniments to the chest—and fomentations and liniments to the abdomen.

Under these cautions and restrictions, we conceive that little danger and much good may arise from attending to Dr. Hall's observations. We have certainly seen several instances where patients were greatly harrassed and reduced by mistakes respecting the existence of internal inflammations; but we never witnessed *fatal* consequences resulting therefrom. We can easily conceive, however, that, in certain situations, as those of parturition, for example, life may be endangered or even destroyed by carrying the mistake too far. We conceive that the thanks of the Profession are due to Dr. Hall for calling their attention to this important topic, and for his endeavours to lay down a diagnostic guide on certain occasions, for the inexperienced practitioner.

### Essay 2.—*On the Effects of Loss of Blood.*

Dr. Hall thinks that the subject of the present Essay, like that of the preceding, has, in a great measure, escaped the attention of the physician and physiologist. But this is too general an expression. That some *particular effects* of loss of blood may have escaped general observation, is very probable; but that the *usual physiological and pathological phenomena* succeeding discharges of the vital fluid, should have eluded the keen observations of many of Dr. Hall's predecessors, is exceedingly unlikely. Who has not observed the syncope—palpitation of the heart—throbbing of the vessels of the head—rapidity of the pulse—violent reaction—dropsical effusions, and many other phenomena succeed to uterine or other hæmorrhages?—Still we are free to confess that Dr. Hall has carried his observations on the *secondary or more remote* effects of sanguineous discharges, farther than any author that we remember to have read. We shall endeavour to follow our author through the various subdivisions into which he has thrown his observations.

I. One of the most common and familiar phenomena resulting from considerable loss of blood, is syncope. This is generally supposed to arise from defect of vigorous circulation through the brain. To vertigo there succeeds loss of consciousness—then a temporary cessation of respiration and circulation. In hæmorrhages, there are various degrees of, or approaches to fainting, the phenomena attending which are familiar to every practitioner. It is well known that when sickness, amounting to vomiting, succeeds, the circulation is improved, and the other symptoms ameliorated.

"In cases of fatal hæmorrhagy there are none of these ameliorations. The symptoms gradually and progressively assume a more and more frightful aspect. The countenance does not improve but becomes more and more pale and sunk; the consciousness sometimes remains until at last there is some delirium; but every thing denotes an impaired state of the energies of the brain; the breathing becomes stertorous and at length affected by terrible gasping; there may be no efforts to vomit; the pulse is extremely feeble or even imperceptible; the animal heat fails and the extremities become colder and colder in spite of every kind of external warmth; the voice may be strong, and there are constant restlessness and jactitation; at length the strength fails, and the patient sinks, gasps, and expires." 40.

But fortunately this is a comparatively rare termination. The system usually recovers itself spontaneously from syncope, by a process which may be termed *reaction*, and which may be either excessive or defective. Or there may be a sinking of the vital powers—each state attended by phenomena peculiar to itself. It may be proper to observe, however, that severe hæmorrhage is not uncommonly productive of another and very different train of symptoms, viz:—of the apoplectic, epileptic, and convulsive class.

II. *Remote, or Cumulative Effects.* In ordinary cases, the recovery or reaction from syncope is a simple return of the functions to their natural state; but after profuse losses of blood, the pulse often acquires and retains a morbid frequency for a certain time, then gradually resumes its natural rhythm. It is otherwise, however, if the person be subjected to repeated blood-lettings, or to a continued drain from the system. In such cases the pulse acquires a preternatural frequency and throbbing beat—and, in some instances, "all the symptoms of

*excessive reaction* of the system," of which Dr. Hall gives the following description.

"The state of excessive reaction is formed gradually, and consists, at first, in forcible beating of the pulse, of the carotids, and of the heart, accompanied by a sense of throbbing in the head, of palpitation of the heart, and eventually perhaps of beating or throbbing in the scrobiculus cordis and in the course of the aorta. This state of reaction is augmented occasionally by a turbulent dream, mental agitation, or bodily exertion. At other times it is modified by a temporary faintness or syncope.

"In the more exquisite cases of excessive reaction the symptoms are still more strongly marked, and demand a fuller description.

"The beating of the temples is at length accompanied by a throbbing pain of the head, and the energies and sensibilities of the *brain* are morbidly augmented; sometimes there is intolerance of light, but still more frequently intolerance of noise and of disturbance of any kind, requiring stillness to be strictly enjoined, the knockers to be tied, and straw to be strewed along the pavement; the sleep is agitated and disturbed by fearful dreams, and the patient is liable to awake or to be awoke in a state of great hurry of mind, sometimes almost approaching to delirium; sometimes there is slight delirium, and occasionally even continued delirium; more frequently there are great noises in the head as of singing,—of crackers,—of a storm,—or of a cataract; in some instances there are flashes of light; sometimes there is a sense of great pressure or tightness in one part or round the head, as if the skull were pressed by an iron nail or bound by an iron hoop.

"The action of the *heart* and *arteries* is morbidly increased, and there are great palpitation, and visible throbbing of the carotids and sometimes even of the abdominal aorta,—augmented to a still greater degree, by every cause of hurry of mind or exertion of the body, by sudden noises or hurried dreams or wakings; the patient is often greatly alarmed and impressed with the feeling of approaching dissolution; the state of palpitation and throbbing are apt to be changed, at different times, to a feeling of syncope; the effect of sleep is in some instances very extraordinary—sometimes palpitation, at other times a degree of syncope, or an overwhelming feeling of dissolution; the pulse varies from 100 to 120 or 130, and is attended with a forcible jerk or bounding of the artery.

"The *respiration* is apt to be frequent and hurried, and attended with alternate panting and sighing; the movement of expiration is sometimes obviously and singularly blended with a movement communicated by the beat of the heart; the patient requires the smelling-bottle, the fan, and fresh air.

"The skin is sometimes hot; and there are frequently general hurry and restlessness.

"In this state of exhaustion, sudden dissolution has sometimes been the immediate consequence of muscular effort on the part of the patient." 44.

This is a faithful description, though the fastidious reader will be apt to pronounce it rather too minute for most practical purposes.

The phenomena of excessive reaction are most observed in young persons of robust constitution, who have been subject to repeated blood-letting. In infants, feeble and aged persons, the reaction is apt to be *defective*.

"In this case the patient remains long pale, thin, and feeble, and becomes faint on the slightest occasions; the pulse is frequent but feeble and perhaps irregular, and we shall look in vain for the throbbing and palpitation observed in the young and robust. This state either gradually yields to returning strength, or subsides into the state of sinking. In the study of the effects of loss of blood it is particularly necessary to bear in mind this difference of the phenomena arising out of the state of the constitution,—of vigour, or of feebleness of the patient." 45.

The symptoms of exhaustion, with excessive reaction, may gradually subside—or they may yield to the state of *sinking*. "This term is adopted not to express a state of *negative weakness* merely, which may continue long, and end in eventual recovery, but to denote a state of positive and progressive failure of the vital powers, attended by its peculiar effects, and by a set of phenomena very different from those of exhaustion with reaction."

In this state of sinking, the functions of the brain, the lungs, and the heart, are said to be singularly impaired.

"The sensibilities of the *brain* subside, and the patient is no longer affected by noises as before; there is, on the contrary, a tendency to dozing, and gradually some of those effects on the muscular system which denote a diminished sensibility of the brain supervene, as snoring, stertor, blowing up of the cheeks in breathing, &c.; instead of the hurry and alarm on awaking as observed in the case of excessive reaction, the patient in the state of sinking, requires a moment to recollect himself and recover his consciousness, is perhaps affected with slight delirium, and he is apt to forget the circumstances of his situation and, inattentive to the objects around him, to fall again into a state of dozing.

"Not less remarkable is the effect of the state of exhaustion with sinking on the function of the *lungs;* indeed the very first indication of this state is, I believe, to be found in the supervention of a *crepitus* in the respiration, only to be heard at first on the most attentive listening; this crepitus gradually becomes more audible and passes into slight rattling, heard in the situation of the bronchiæ and trachea; there is also a degree of labour or oppression in the breathing, inducing acuteness in the nostrils, which are dilated below and drawn in above the lobes at each inspiration; in some cases there is besides, a peculiar catching, laryngeal cough, which is especially apt to come on during sleep, and awakes, or imperfectly awakes the patient.

"The *heart* has, at the same time, lost its violent beat and palpitation, and the pulse and arteries their bounding or throbbing.

"The stomach and bowels become disordered and flatulent, and the command over the sphincters is impaired.

"The last stage of sinking is denoted by a pale and sunk countenance, inquietude, jactitation, delirium, and coldness of the extremities." 47.

The symptoms of exhaustion, reaction, and sinking are strikingly illustrated by the melancholy case of a medical gentleman which occurred, we believe, under the roof of our intelligent author. We shall present an abstract of this case to our readers.

*Case.* Mr. C. aged 40, very robust and muscular, was thrown under his horse on the 3d of October, 1821, and had his third and fourth ribs, of the left side, fractured. He was seen early next morning by our author and a surgeon. There was distinct crepitus, but no emphysema—there was much pain—pulse 100, and strong. V. S. to 16oz. a dozen of leeches to the temples—and the same number over the fractured ribs. The motions of the ribs restrained by a proper bandage—mercurial and other purgatives. At noon another venesection to 16oz. During the whole of October 5, the patient appeared to be doing well; but in the night, a violent attack of pain in the side induced the patient to bleed himself to syncope. Seventeen leeches to the side. Great relief was thus obtained—purgation continued. 6th. Early this morning, another violent attack of pain, with dyspnœa. Mr. C. again bled himself, to 24 ounces, and to syncope. Great relief. 7th. Removed in a litter to Dr. Hall's house. Evening, an increase of pain and a small bleeding. The patient had now lost, in all, about 120 ounces of blood. 8th. An eminent physician and surgeon were added in consultation. Much pain of side, and venesection was again proposed; but objected to by Dr. Hall, as he had observed some symptoms indicative of reaction from exhaustion. The symptoms increased. 9th. (or 6th day from the receipt of the injury) There was some dyspnœa, and pain of the side—pulse 100, with a peculiar jerk—violent throbbing of the carotids—pulsatory pain in the head—intolerantia lucis et soni—agitation when a sudden noise was made at the door. The bowels having been freely opened, a draught with tinct. opii et spir. ammon. aromat. was given, together with broth and other light nutriment. 10th. All the symptoms of reaction continue—head relieved by a cold lotion. 11th. Pulse reduced to 84, and had lost some of the jerk—the carotids beat less violently—there is more tranquillity and hilarity of mind. The aperients, anodyne, and nourishment continued. 12th. Dr. Hall saw his patient at 3 o'clock in the morning of this, the ninth, day, and he then heard a slight degree of that crepitus in the breathing, which he considers as an early symptom of *sinking*. At nine o'clock the consultation was assembled, but the other medical gentlemen did not see any cause for alarm. Dr. H. mentioned his fears. Cupping was proposed, but the changes for the worse were so rapid, that brandy was recommended in the evening. In the course of the day the carotid throbbing had subsided—a slight degree of stupor came on, with some labour in respiration, a dry cough, anxiety of countenance, but no pain in the side. In the course of the night the patient became insensible, and expired a little after 3 o'clock in the morning.

On dissection the pleura was found morbidly red in the vicinity of the fracture, but not wounded. There was some effusion of lymph in its cavity. These are all the particulars of the dissection given. It is a great pity that the state of the brain is not particularly described.

Although it is probable that this unfortunate gentleman lost too much blood, and that his death was owing to this over-depletion; yet we do not see that it could well have been avoided, since the symptoms appeared to indicate the measure, and relief always followed the depletion. The case will, therefore, prove a caution, rather than a guide, on future occasions.

III. *On the Effects of further loss of Blood in Cases of Exhaustion.* Dr. H. is convinced that the symptoms of exhaustion with reaction have been frequently mistaken for inflammation, under which impression the lancet has been repeatedly employed, and the deception kept up—"for all the symptoms are, perhaps, fully relieved."—This was a puzzling fact.

"At length I discovered, by a careful observation that the symptoms which were relieved were those of reaction; and that the mode of relief was by the substitution of syncope; that the relief endured as long as the state of faintishness continued, but returned as this state gave way to the rallying and reaction of the vital powers.

"Another circumstance equally interesting and curious was, that within certain limits, the remedy which relieved for a time, eventually only added to the severity of the malady, for that this was apt to return after a certain period, in a still more aggravated form. It is natural, indeed, to suppose that, unless there was a tendency to the failure of the vital powers, the reaction of the system and the painful circumstances attending it, would be greater after a third or fourth loss of blood, than after a first or second; indeed there are seldom the symptoms of reaction after one flow of blood, however great or profuse; it is the repetition or protraction of the cause which, as I have already observed, is essential to produce this effect." 58.

It is observable too, that, in cases of exhaustion with reaction, syncope is very soon produced by the further loss of blood —a fact of importance, as a *sign* of the state of exhaustion, and as a warning voice against further and inconsiderate depletion.

If the loss of blood be carried still farther, a state of sinking is induced, and all reaction disappears.

There are various circumstances which influence the effects of loss of blood—the principal one of which is the *strength* of the patient. Cæteris paribus, the reaction will be in proportion to this strength. In infancy—in declining life—and in feeble constitutions, there is *defective reaction* after loss of blood, and the state of syncope is a state of danger. In youth, and in robust constitutions, on the contrary, the reaction is strong, and especially marked after repeated venesections. In the vigorous, the state of sinking is always preceded by great reaction, unless the strength be completely overwhelmed by the extent or the repetition of the early depletion. In the feeble, it steals on insidiously, unmarked by reaction of the system.

Intestinal irritation is another circumstance which exerts a conspicuous influence on the effects of sanguineous losses—in contra-distinction to inflammation, which seems to protect the system from the effects of loss of blood. In the *former* case, the throbbing is soon induced—in the *latter*, blood-letting is followed by little of reaction until the state of inflammation be subdued, and the system be freely exposed to the uncontrolled influence of loss of blood.

" In all cases we are only to expect the phenomena of reaction when a *certain quantity* of blood has been lost; one bleeding, although large, and even a continued drain, if not considerable, will not induce exhaustion; the powers of the system are sufficiently great to *recruit* and to *restrain* its actions.—But exhaustion is sooner induced under circumstances of intestinal irritation, and less so under those of inflammation than in health, and reaction is the consequence, unless the strength of the patient be low, and then the reaction is defective, or even gives way to a state of positive sinking." 67.

Each successive blood-letting is, of course, attended with increasing risk. There is considerable danger where the reaction is strong, but still more when it is feeble. A large blood-letting in such cases may be followed by sudden death. There is great danger when fainting has been several times induced, and when there is the least tendency to —" want of fresh air."

Our limits have been so far overstepped that we must pass unnoticed, the last Essay, "on sinking and exhaustion." It is not, however, of near so much interest or importance as the essays preceding it. Dr. Hall would find some interesting information on "the effects of loss of blood on the internal organs," in Dr. Seeds' Thesis, in which a number of experiments were made on dogs and other animals, by bleeding them to death. In almost all the instances, there were effusions of serum or even of blood in the brain. The heart and large vessels were sometimes found gorged with blood, as if the remains of the vital fluid were concentrated in excess around the citadels of life, when the rest of it was drained off from the system. Vialle, in his Essay or Super and Sub-excitement, also states some curious facts bearing on this subject.

But here we must conclude, wishing our ingenious and talented author every success in his zealous researches into the phenomena of disease.

